# Dose‐related cardiac outcomes in response to postnatal dexamethasone treatment in premature lambs

**DOI:** 10.1002/ar.25202

**Published:** 2023-03-16

**Authors:** Amanda Vrselja, Jennifer Jane Pillow, Jonathan G. Bensley, Siavash Ahmadi‐Noorbakhsh, Peter B. Noble, Mary Jane Black

**Affiliations:** ^1^ Department of Anatomy and Developmental Biology, Monash Biomedicine Discovery Institute Monash University Clayton Victoria Australia; ^2^ School of Human Sciences University of Western Australia Perth Western Australia Australia

**Keywords:** cardiac remodeling, developmental programming, infant, premature, myocardium, myocyte, cardiac, postnatal corticosteroids, premature birth

## Abstract

**Background:**

Postnatal corticosteroids are used in the critical care of preterm infants for the prevention and treatment of bronchopulmonary dysplasia. We aimed to investigate the effects of early postnatal dexamethasone therapy and dose on cardiac maturation and morphology in preterm lambs.

**Methods:**

Lambs were delivered prematurely at ~128 days of gestational age and managed postnatally according to best clinical practice. Preterm lambs were administered dexamethasone daily at either a low‐dose (*n* = 9) or a high‐dose (*n* = 7), or were naïve to steroid treatment and administered saline (*n* = 9), over a 7‐day time‐course. Hearts were studied at postnatal Day 7 for gene expression and assessment of myocardial structure.

**Results:**

High‐dose dexamethasone treatment in the early postnatal period led to marked differences in cardiac gene expression, altered cardiomyocyte maturation and reduced cardiomyocyte endowment in the right ventricle, as well as increased inflammatory infiltrates into the left ventricle. Low‐dose exposure had minimal effects on the preterm heart.

**Conclusion:**

Neonatal dexamethasone treatment led to adverse effects in the preterm heart in a dose‐dependent manner within the first week of life. The observed cardiac changes associated with high‐dose postnatal dexamethasone treatment may influence postnatal growth and remodeling of the preterm heart and subsequent long‐term cardiac function.

## 1 INTRODUCTION

Preterm birth (<37 weeks of gestation) is strongly associated with adverse neonatal health outcomes, and these short‐ and long‐term outcomes depend on the degree of prematurity. The premature interruption to organogenesis coupled with the adaptive changes in the neonatal period potentially program an increased susceptibility to disease later in life. The fetal heart is vulnerable to change during late gestation because cardiomyocytes undergo a maturational transition during this period: the cardiomyocyte population progressively loses its proliferative capacity and the cells become differentiated (Burrell et al., 2003; Clubb Jr. & Bishop, 1984; Thornburg et al., 2011). Consequently, a high proportion of cardiomyocytes are immature at birth in the preterm heart. There is clear evidence associating preterm birth with structural cardiac remodeling and impaired function by early adulthood (Lewandowski et al., 2013a; Lewandowski et al., 2013b; Lewandowski et al., 2021; Mohamed et al., 2020) as well as an increased risk of early heart failure and ischemic heart disease (Carr et al., 2017; Crump et al., 2019; Crump et al., 2021). Early life events associated with the postnatal care of preterm infants may further critically influence cardiomyocyte growth and endowment in the neonatal period and thus lead to long‐term programming of cardiac structure and function (Barker, 2004; Gluckman & Hanson, 2004; Jonker & Louey, 2016).

Antenatal corticosteroids and intensive neonatal care practices play a crucial role in assisting a viable transition from fetal to postnatal life following premature delivery and hence facilitate the survival of the most vulnerable preterm infants, those born very (<32 weeks of gestational age,) and extremely (<28 weeks of gestational age) preterm. Very and extremely preterm infants often require ventilation to support breathing but they can become ventilator dependent and as a result, are susceptible to developing bronchopulmonary dysplasia. Glucocorticoids are administered postnatally to facilitate extubation from respiratory support and for the treatment of severe bronchopulmonary dysplasia. The potent anti‐inflammatory properties of glucocorticoids can downregulate the persistent pulmonary inflammation (Bolt et al., 2001; Jobe, 2009). Unfortunately, however, the postnatal administration of corticosteroids is not devoid of harmful side effects and therefore remains a highly contentious clinical practice (Gupta et al., 2012; Watterberg, 2012).

Preterm infants treated postnatally with glucocorticoids (usually dexamethasone) often develop hypertrophic cardiomyopathy; characterized as hypertrophy of the left ventricle (LV) and interventricular septum (Evans, 1994; Gill et al., 1996; Werner et al., 1992; Zecca et al., 2001). Interestingly, the cardiac hypertrophy resolves shortly after the infant is weaned off dexamethasone (Evans, 1994; Skelton et al., 1998; Zecca et al., 2001). Experimental animal models consistently report transient cardiac hypertrophy immediately following postnatal dexamethasone treatment (De et al., 2011; LaMear et al., 1997; Liu et al., 2007; Muangmingsuk et al., 2000; Sicard & Werner, 1992). However, whether there are persistent alterations in cardiac structure and cardiomyocyte growth following this transient early life event remains unclear. Historically, many infants (that are now in adulthood) were treated with high‐doses of postnatal corticosteroids while undergoing neonatal intensive care, although the high‐dose regimen is no longer used clinically. In the present study, we utilized a preclinical model of premature birth in lambs to investigate the effects of postnatal treatment of a clinically relevant high‐ and low‐dose of dexamethasone on cardiac structure and maturation. We hypothesized that dexamethasone treatment results in adaptive remodeling of the immature preterm heart and that there is a dose‐related acceleration of cardiomyocyte maturation and cardiac remodeling effect. The aim was to determine whether early postnatal administration of dexamethasone independently alters cardiac structure, gene expression, immune cell infiltration, and cardiomyocyte maturation and growth (cell cycle activity, nuclearity, size, and endowment) in the preterm heart, and if so, whether there is a dose effect.

## 2 METHODS

### 2.1 Ethics approval

The Animal Ethics Committee of the University of Western Australia approved all animal experimentation (RA 3/100/1301) in accordance with the Australian code for the care and use of animals for scientific purposes.

### 2.2 Preterm delivery

Pregnant ewes received intramuscular (IM) medroxyprogesterone (150 mg; Depo‐Provera, Pfizer, Australia) 7 days prior to their scheduled preterm delivery, to avoid betamethasone‐induced premature labour. Ewes were administered maternal IM betamethasone (5.7 mg/2 ml; Celestone Chronodose; Merck Sharp & Dohme Pty Ltd., NSW, Australia) at 48 and 24 hr before preterm delivery. Caesarean delivery of preterm lambs occurred at ~128 days of gestational age (term is ~150 days of gestational age). Prior to delivery, fetuses were catheterized, intubated, and administered surfactant (3 ml, 80 mg/ml, poractant alfa: Chiesi Farmaceutici S.p.A., Parma, Italy). Lambs were weighed, manually resuscitated and then mechanically ventilated. Contemporary ventilation monitoring was based on information obtained from the ventilator (Babylog VN500 Ventilator, Dräger Medical, Lübeck, Germany), with the aim to gradually reduce respiratory support to unassisted breathing (Papagianis et al., 2021). Preterm lambs were managed postnatally with 24‐hr continuous postnatal care and monitoring in accordance with contemporary clinical management, including observation of heart rate, using a neonatal pulse oximeter gently secured around an ear, and blood pressure monitoring, through the arterial catheter connected to the arterial transducer and noninvasively using a sphygmomanometer.

Lambs were intravenously administered dexamethasone (dexamethasone sodium phosphate; Aspen Pharmacare Australia Pty Ltd., St Leonards, NSW, Australia) daily at either a tapered low‐dose (Preterm LD, *n* = 9; 0.15 mg/kg/days × 72 hr followed by 0.1 mg/kg/days × 48 hr and then 0.05 mg/kg/days × 48 hr) or a tapered high‐dose (Preterm HD, *n* = 7; 0.5 mg/kg/days × 72 hr followed by 0.3 mg/kg/days × 72 hr and then 0.27 mg/kg/days × 24 hr) or were naïve to postnatal steroid treatment (preterm SAL, *n* = 9; administered saline over a similar time‐course). The outcomes for preterm lambs treated with saline and naïve to dexamethasone treatment were reported previously (Vrselja et al., 2022); data from these lambs are used in the present study for comparison as a nonsteroid postnatal treatment group.

Preterm lambs were humanely killed at 7 days of postnatal age with an overdose of sodium pentobarbitone (150 mg/kg; Jurox Pty Ltd., Australia). At post‐mortem, hearts were excised, weighed and ventricle tissue sampled (1 cm^3^) from the free wall of the right and left ventricles (LVs) and snap frozen for molecular analyses. The hearts were then retrogradely perfusion fixed.

### 2.3 Heart sampling and analysis

The atria were removed from the perfusion‐fixed hearts and the ventricles transversely sliced into 10‐mm thick sections. The LV and interventricular septum (LV + S) were separated from the RV and the LV + S and RV were independently sampled using a smooth fractionator approach (Gundersen, 2002; Stacy et al., 2009); 8–10 pieces were sampled per ventricle and embedded in glycolmethacrylate or paraffin.

### 2.4 Quantitative assessment of myocardial collagen content

Paraffin‐embedded LV + S and RV sections pre‐treated with Bouin's fixative were stained with picrosirius red for the assessment of myocardial collagen content. Whole slide scans (Aperio AT Turbo Scanner, Leica Biosystems, Vista, CA) were opened in Aperio Imagescope (Version 12.3.3, Leica Biosystems, Vista, CA) for automated quantification of interstitial collagen content (Bensley et al., 2018), perivascular collagen was excluded from analyses. A customized Positive Pixel Count algorithm in Aperio Imagescope analyzed interstitial collagen content in approximately 50–75 fields of view (339,690 μm^2^) randomly sampled at 20× magnification. Collagen content was expressed as a percentage of the area of collagen divided by the total area of tissue.

### 2.5 Quantitative assessment for myocardial immune cell infiltration and cardiomyocyte cell cycle activity

Paraffin‐embedded LV + S and RV sections were stained immunohistochemically for the assessment of myocardial immune cell infiltration, using a CD45 (common leukocyte antigen) antibody, and cell cycle activity, using a Ki67 antibody, using a DAKO Austostainer Plus staining system, as described previously (Flores et al., 2018; Vrselja et al., 2022). All immunohistochemical stained slides were whole scanned at 40× magnification using an Aperio AT Turbo Scanner (Leica Biosystems, Vista, California, USA) and images exported to Aperio Imagescope for analyses.

Myocardial immune cell infiltration (in CD45 immunohistochemically stained sections) was assessed in randomly selected regions of interest (13,046 μm^2^) for more than 50 separate fields of view per ventricle. The number of positive CD45 cells were manually assessed in each field of view and the average number of positive CD45 cells per unit of area calculated.

Cardiomyocyte cell cycle activity (in Ki67 immunohistochemically stained sections) was assessed within clearly defined inclusion and exclusion areas using an optimized nuclear count algorithm in the program Aperio Imagescope. To ensure cardiomyocytes were distinguished from fibroblasts and other noncardiomyocyte cells, images were modified by manually drawing inclusion and exclusion zones on each image to exclude positive Ki‐67 noncardiomyocyte cells (Bensley et al., 2018; Vrselja et al., 2022). The nuclear count algorithm was optimized to count for positively Ki67 stained cardiomyocyte nuclei based on their morphometry by factoring and optimizing for minimum and maximum nuclear size, roundness and elongation of the nucleus and staining threshold. The modified images were processed for the quantification of all cardiomyocyte nuclei and the proportion of Ki67‐positive cardiomyocyte nuclei.

### 2.6 Quantitative assessment of cardiomyocyte nuclearity and cross‐sectional area

Cardiomyocyte nuclearity and cross‐sectional area in the LV + S and RV were visualized in long axis or short axis orientation, respectively, and quantified in 5 μm paraffin sections stained with Wheat Germ Agglutinin (WGA) conjugated to Alexa‐Fluor 488 (Invitrogen, USA), and DAPI (4′,6‐diamidino‐2‐phenylindole) (Invitrogen, USA), as previously described (Bensley et al., 2016; Vrselja et al., 2022). Cardiomyocytes in the long axis orientation were visualized to quantify nuclearity in more than 250 cardiomyocytes per ventricle per animal (Bensley et al., 2010). Cardiomyocyte cross‐sectional area (a proxy for cell size) was measured in short axis orientation, with a centrally located nucleus, in more than 300 cardiomyocytes per ventricle per animal and quantified using image analysis (Fiji, Version 2.0.0) (Goh et al., 2011).

### 2.7 Stereological estimation of cardiomyocyte number

Glycolmethacrylate‐embedded tissue was sectioned at 20‐μm thick and every 40th section was collected and stained with Harris' hematoxylin (Amber Scientific, Queensland, Australia) for identification of nuclei. An optical disector‐fractionator approach was used to stereologically estimate the total number of cardiomyocyte nuclei (Bensley et al., 2010;Bruel & Nyengaard, 2005; Corstius et al., 2005). Uniform systematic sampling (step length of 2,000 μm) commenced at a random point and counting was conducted within a superimposed unbiased counting frame (538.4 μm^2^). Cardiomyocyte nuclei were counted if present within the disector area of the counting frame and nuclei were not touching the exclusion zones of the counting frame and in clear focus in the middle 10 μm of the section. Total number of cardiomyocyte nuclei were estimated in the walls of the LV + S and RV. The total number of cardiomyocytes was determined subsequently taking into account cardiomyocyte nuclearity (Bruel & Nyengaard, 2005; Corstius et al., 2005).

### 2.8 Gene expression

Total RNA was purified from LV and RV tissue using the PureLink™ RNA Mini Kit (Invitrogen, Life Technologies, Carlsbad) with TRIzol® Reagent (Invitrogen, Life Technologies, Carlsbad) according to manufacturer's instructions. The SuperScript® III First‐Strand Synthesis System (Invitrogen, Life Technologies, Carlsbad, CA) was used to synthesize cDNA from purified total RNA; a negative control, –Reverse Transcriptase (−RT) control, was prepared for quality checking of samples.

TaqMan® probes (Applied Biosystems, Life Technologies, Carlsbad, CA) were used to measure genes of interest expression. The target genes specifically related to angiogenesis (*VEGFA*), calcium handling (*ATP2A2*, *RYR2*, and *SLC8A1*), collagen synthesis (*COL1A1* and *COL3A1*), extracellular matrix modulation (*TIMP2*), inflammation (*IL‐1b*, *IL‐18*, *TGFβ1*, *TLR4*, and *TNF*) and cardiac hypertrophy (*GATA4*, *IGF‐1*, *MYH7*, and *NPPA*), metabolism (*PPARA* and *PPARGC1A*) and remodeling (*MMP9*).

All samples, cDNA and –RT control samples, were quality checked by SYBR™ chemistry qPCR for gDNA contamination using the 7900HT Fast real‐time PCR system (Applied Biosystems, Life Technologies, Carlsbad, CA). The Biomark™ HD (Fluidigm, South San Francisco, CA) dynamic integrated fluidic circuit was used to analyze gene expression, via real‐time PCR using the 7900HT fast real‐time PCR systems. Ct values were analyzed using the qBase+ software (version 3.1; Biogazelle, Gent, Belgium) (Hellemans et al., 2007). Gene expression was normalized to *YWHAZ* and expressed relative to the preterm saline (control) group (values were normalized to 1.00), known as the calibrated normalized relative quantity (CNRQ).

### 2.9 Statistical analyses

Statistical analyses were conducted using GraphPad Prism (Version 7.0b, GraphPad Software, USA). Data were checked for normality then analyzed by a one‐way ANOVA followed by a Tukey post hoc test. Gene expression analyses were conducted in qBase+ software (version 3.1; Biogazelle, Gent, Belgium) using a one‐way ANOVA followed by a Tukey–Kramer post hoc test. Data are represented as the mean ± *SD* and significance was accepted at *p*‐value <.05.

## 3 RESULTS

### 3.1 Body weights and heart weights

Body weight and heart weight measurements are summarized in Table 1. All lambs were delivered preterm at ~128–129 days gestational age and were similar in birth weight. All preterm lambs had a decreased body weight at necropsy. Mean heart weights at necropsy were not significantly different between the preterm low‐dose dexamethasone treated and control lambs. However, mean heart weight of the preterm lambs treated with high‐dose dexamethasone was significantly reduced (*p* = .047) compared to the control preterm group. There was no difference in the relative heart weight between all groups, when heart weight was adjusted to body weight.

**TABLE 1:** Characteristics of the preterm lambs and their hearts at delivery and necropsy.

	Preterm SAL	Preterm LD	Preterm HD
Gestational age (days)	129.7 ± 1.4	128.4 ± 1.0	128.3 ± 0.5
N (male)	9 (4)	9 (7)	7 (4)
Birth body weight (kg)	2.90 ± 0.41	3.23 ± 0.40	2.98 ± 0.36
Necropsy body weight (kg)	2.79 ± 0.39	2.75 ± 0.29	2.67 ± 0.47
Heart weight (g)	23.7 ± 3.1	23.2 ± 2.2	20.5 ± 2.1*
Heart weight: body weight at necropsy (g/kg)	8.64 ± 1.61	8.45 ± 0.74	7.86 ± 1.38

*Note*: Data are presented as mean ± *SD*. Significance accepted at *p* < .05.

* Significant difference compared to preterm SAL.

### 3.2 Mean blood pressure and heart rate

Mean arterial blood pressure and heart rate at postnatal Days 1 and 7 are summarized in Table 2. Mean arterial blood pressure was similar between all preterm lambs on the day of birth (Day 1) and at 1 week of postnatal life. However, preterm lambs treated with high‐dose dexamethasone had lower heart rates compared to control lambs at postnatal Day 1 (*p* = .0073) and Day 7 (*p* = .019). The heart rate was also significantly reduced compared to low‐dose dexamethasone treatment at postnatal Day 7 (*p* = .026).

**TABLE 2:** Mean arterial blood pressure and heart rate of the preterm lambs on the day of birth and at 1 week of life.

		Preterm SAL (*n* = 9)	Preterm LD (*n* = 9)	Preterm HD (*n* = 7)
Arterial blood pressure (mmHg)	Day 1	62.8 ± 5.0	61.3 ± 5.8	57.0 ± 3.8
	Day 7	64.5 ± 4.7	66.4 ± 7.5	66.2 ± 3.6
Heart rate (beats/min)	Day 1	220.2 ± 17.0	202.8 ± 13.6	194.7 ± 13.9*
	Day 7	216.6 ± 29.6	214.5 ± 26.2	177.1 ± 21.7* ^,^ **

*Note*: Data are presented as mean ± *SD*. Signifiance accepted at *p* < .05.

* Significant difference compared to preterm SAL.

** Significant difference compared to preterm LD.

### 3.3 Myocardial interstitial collagen content

**FIGURE 1:**
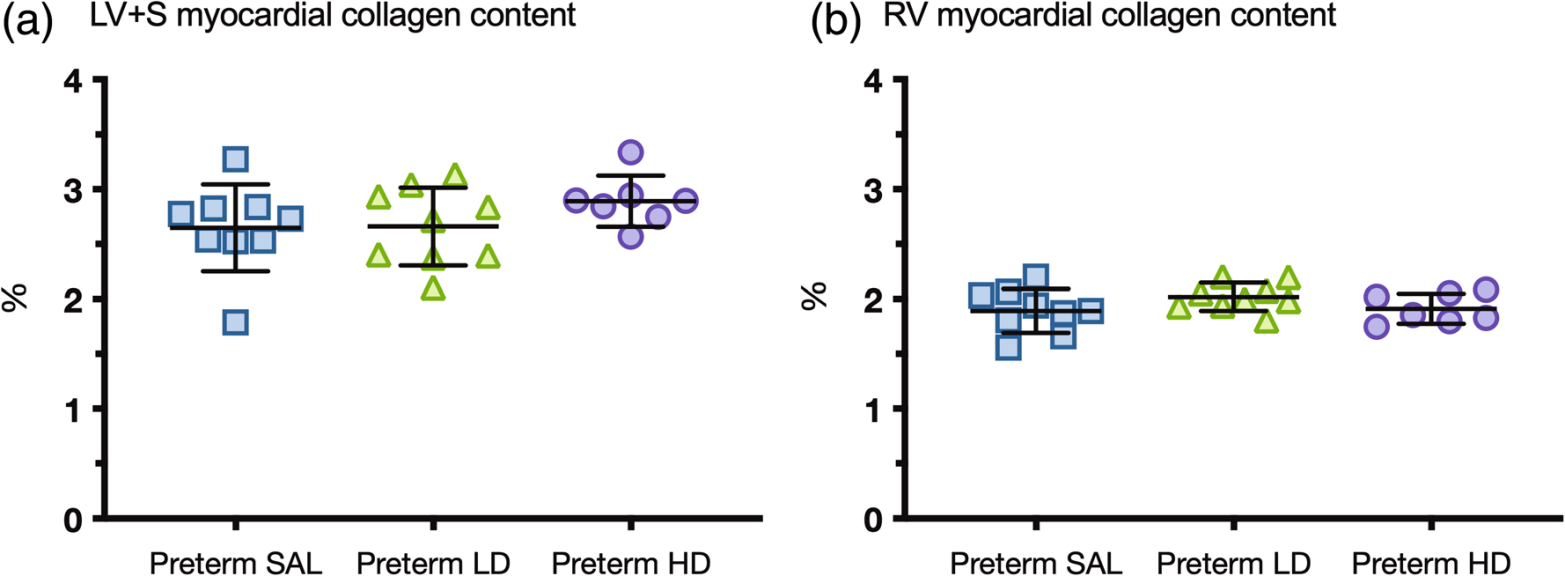
Interstitial myocardial collagen content of preterm hearts. Interstitial myocardial collagen content was assessed in the left ventricle + septum (LV + S; a) and right ventricle (RV; b) of preterm lambs treated with saline (blue squares, preterm SAL, *n* = 9) or dexamethasone at a low‐dose (green triangles, Preterm LD, *n* = 9) or high‐dose (purple circles, preterm HD, *n* = 7). Data are presented as mean ± *SD*.

The overall interstitial collagen content of the preterm hearts was lower in the RV compared to the LV + S in all groups (Figure 1). The LV + S myocardial collagen content was similar between all preterm lambs. The RV myocardial collagen content was also similar between all preterm lambs.

### 3.4 Myocardial immune cell infiltration

**FIGURE 2:**
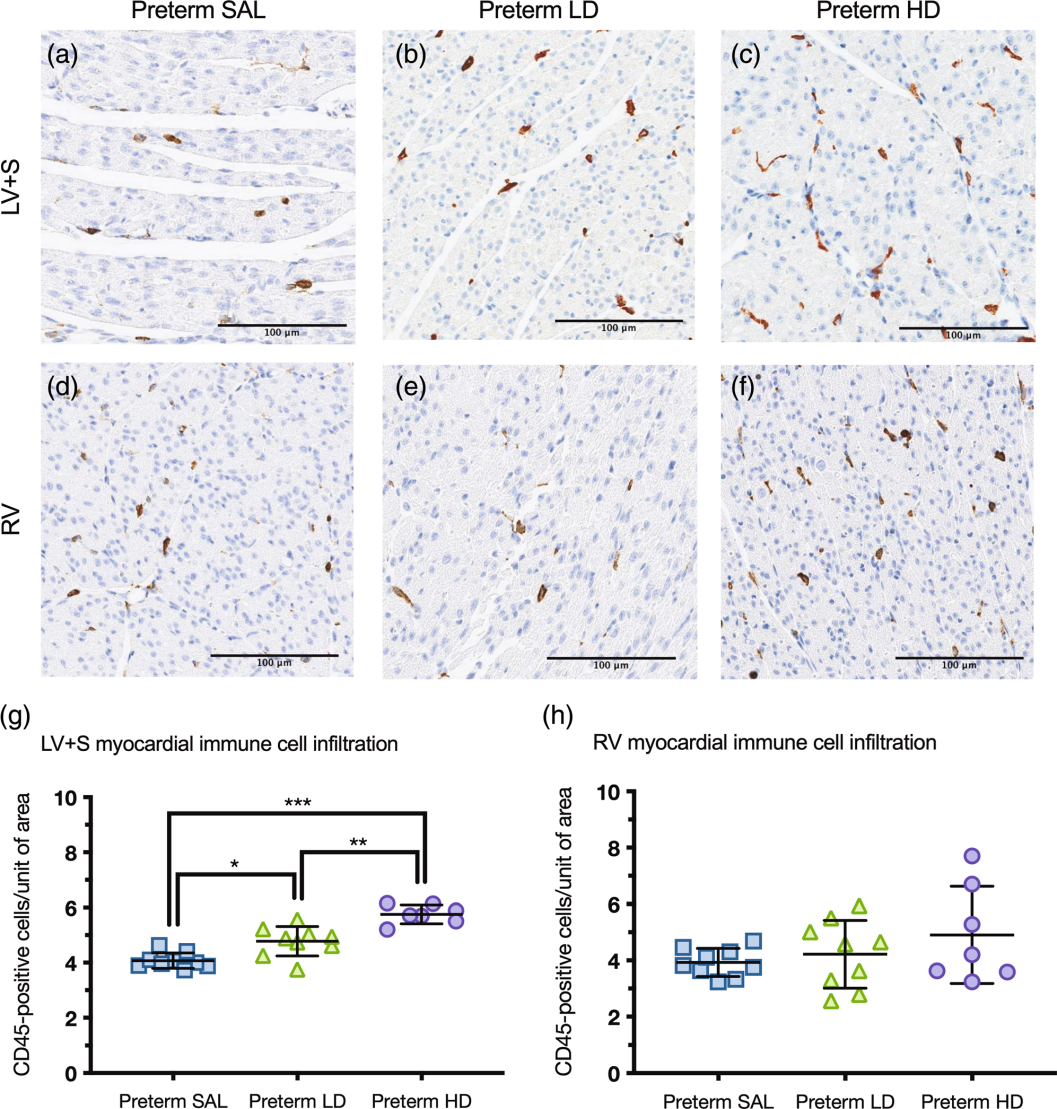
Myocardial immune cell infiltration. Representative images of CD45 immunohistochemical stained myocardium from the left ventricle + septum (LV + S; a–c) and right ventricle (RV; d–f) of preterm lambs treated with saline (preterm SAL, *n* = 9) or dexamethasone at a low‐dose (preterm LD, *n* = 9) or high‐dose (preterm HD, *n* = 7). Scale bar = 100 μm. The number of positive CD45 cells per unit of area was analyzed in the left ventricle + septum (g) and right ventricle (h) for each experimental group (blue squares = preterm SAL; green triangles = preterm LD; purple circles = preterm HD). Data are presented as mean ± *SD*, *p < .05, **p < .001, *** *p* < .0001.

Myocardial immune cell infiltration, as detected by positive CD45 cells, was increased in the LV + S of preterm lambs administered postnatal dexamethasone following either a low‐dose (*p* = .0036) or high‐dose (*p* < .0001) compared to preterm saline‐treated lambs (Figure 2). Increased immune cell infiltrates within the LV + S in lambs administered postnatal dexamethasone were greater in the high‐dose treatment group (*p* = .0002) compared to the low‐dose treatment group. In contrast, the immune cell infiltrates present in the RV were unaffected by postnatal steroid treatment.

### 3.5 Cardiomyocyte cell cycle activity and maturation

**FIGURE 3:**
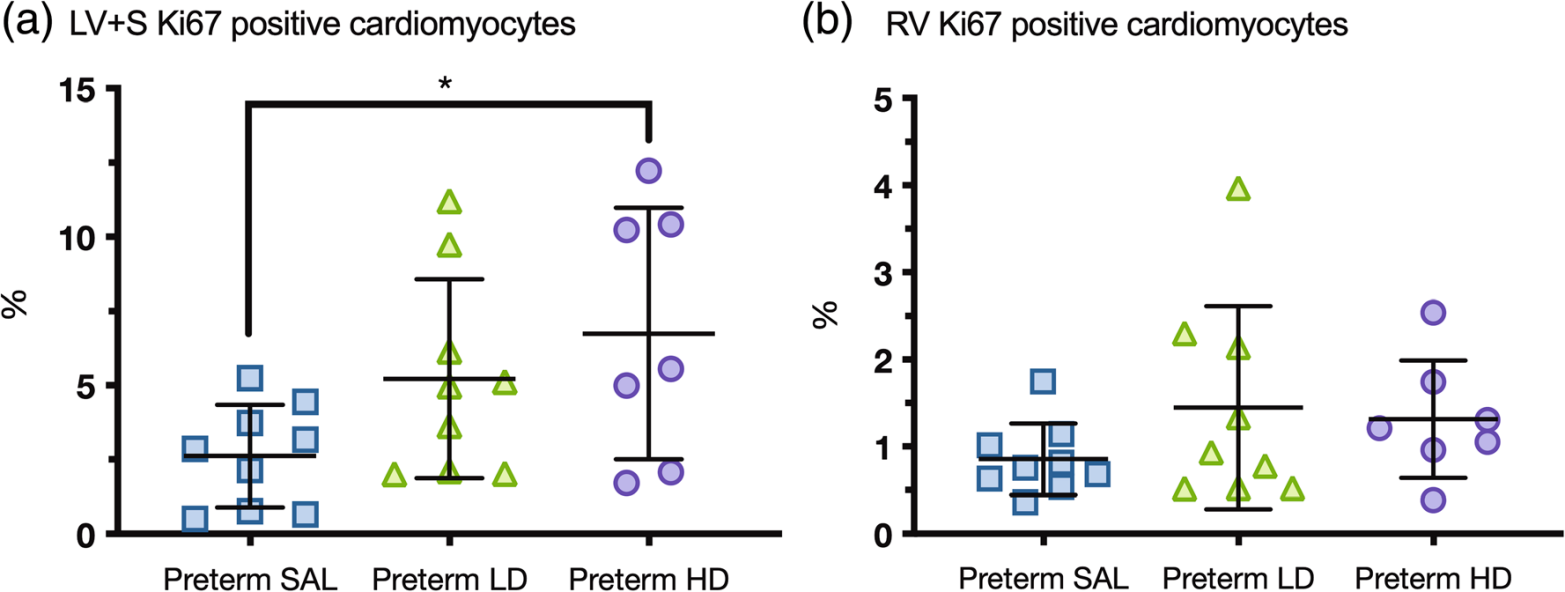
Cardiomyocyte cell cycle activity. The proportion of positive Ki67 cardiomyocyte nuclei in the left ventricle + septum (LV + S, a) and right ventricle (RV, b) was analyzed in each of the experimental groups (blue squares = preterm SAL; green triangles = preterm LD; purple circles = preterm HD). Data are presented as mean ± *SD*, *p < .05.

Preterm lambs treated with high‐dose dexamethasone showed increased cardiomyocyte cell cycle activity within the LV + S compared to the control preterm saline group, as indicated by the greater proportion of positively stained Ki67 cardiomyocytes (*p* = .043) (Figure 3). In contrast, preterm lambs treated with low‐dose dexamethasone showed no significant difference in cardiomyocyte cell cycle activity within the LV + S compared to the control saline group (*p* = .21). Cardiomyocyte cell cycle activity in the RV was low and similar between all groups.

**FIGURE 4:**
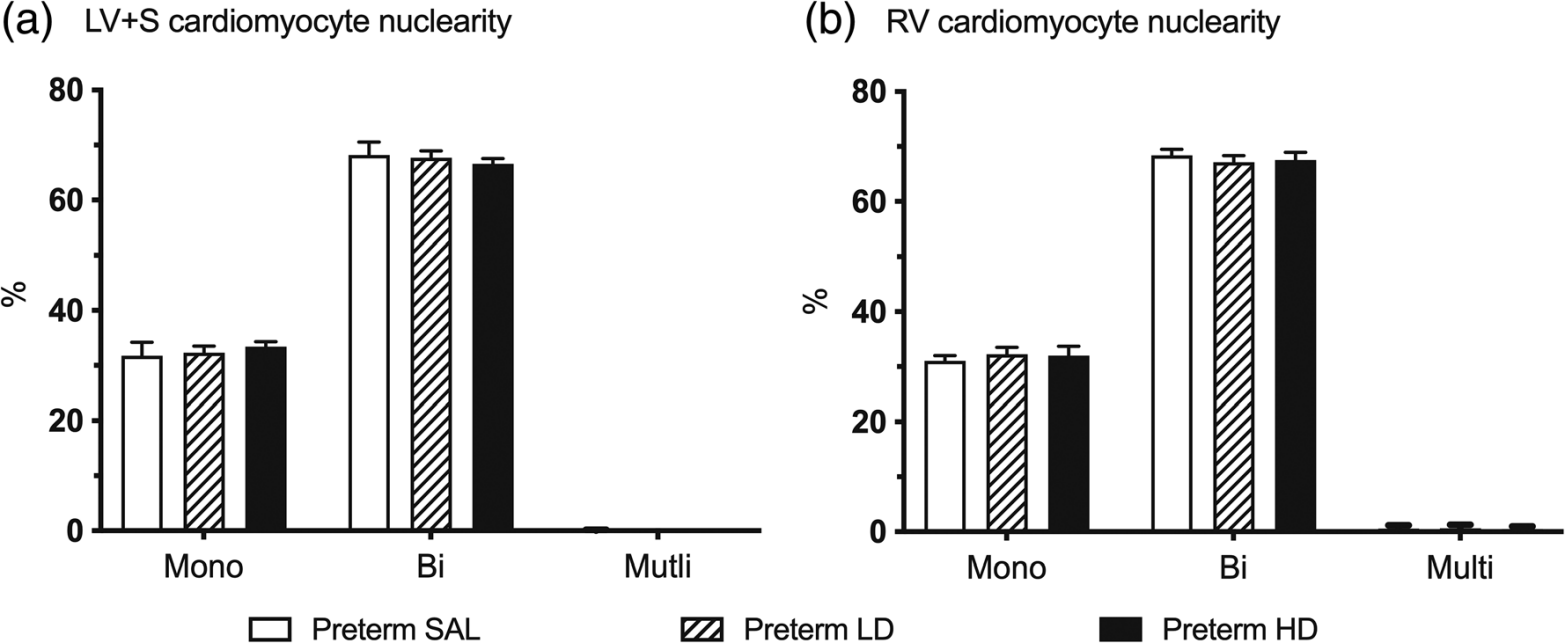
Cardiomyocyte nuclearity. The proportions of cardiomyocyte nuclearity (mono‐nucleated, bi‐nucleated or multi‐nucleated) was analyzed in the left ventricle + septum (LV + S; a) and right ventricle (RV; b) of preterm lambs treated with saline (white bars, Preterm SAL, *n* = 9) or dexamethasone at a low‐dose (striped bars, Preterm LD, *n* = 9) or high‐dose (black bars, Preterm HD, *n* = 7). Data are presented as mean ± SEM.

The proportion of cardiomyocyte nuclearity (mono‐, bi‐, tri‐, and tetra‐nucleated) within the LV + S and RV was similar in all groups. All preterm lambs had similar proportions of mononucleated and binucleated cardiomyocytes in the LV + S (Figure 4). The same was observed in the RV, with similar proportions of mononucleated and binucleated cardiomyocytes. Multinucleated (>2 nuclei) cardiomyocytes were present in both ventricles but no significant differences between groups.

### 3.6 Cardiomyocyte endowment

**FIGURE 5:**
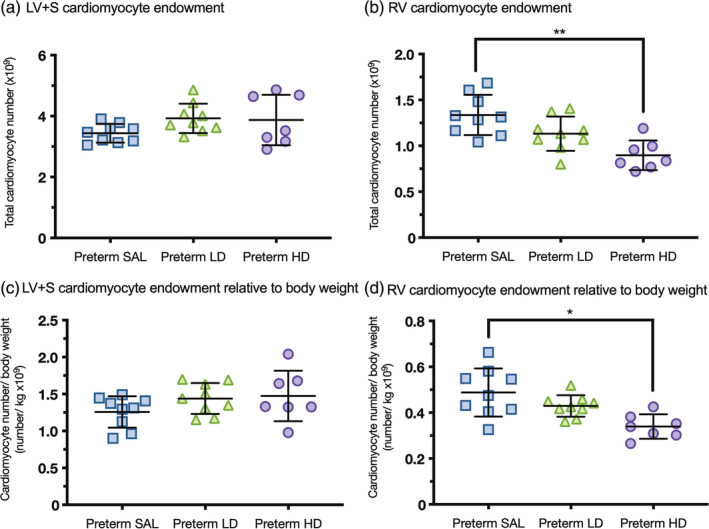
Cardiomyocyte endowment. Cardiomyocyte number was assessed in the left ventricle + septum (LV + S; a) and right ventricle (RV; b) and normalized to body weight (c and d) of preterm lambs treated with saline (blue squares, preterm SAL, *n* = 9) or dexamethasone at a low‐dose (green triangles, Preterm LD, *n* = 9) or high‐dose (purple circles, Preterm HD, *n* = 7). Data are presented as mean ± *SD*, *p < .05, **p < .001.

Postnatal dexamethasone treatment did not influence the total cardiomyocyte number of the LV + S nor when relative to body weight (Figure 5). In contrast, preterm lambs treated with high‐dose dexamethasone (*p* = .0005) had a reduced complement of cardiomyocytes in the RV compared to control preterm lambs. The reduced complement of RV cardiomyocytes remained significantly reduced when expressed relative to body weight (*p* = .0019) in preterm lambs treated with high‐dose dexamethasone compared to the control preterm lambs. There was a nonsignificant trend towards a reduced complement of RV cardiomyocytes in preterm lambs treated with low‐dose dexamethasone (*p* = .061) compared to control preterm lambs.

### 3.7 Cardiomyocyte cross‐sectional area

**FIGURE 6:**
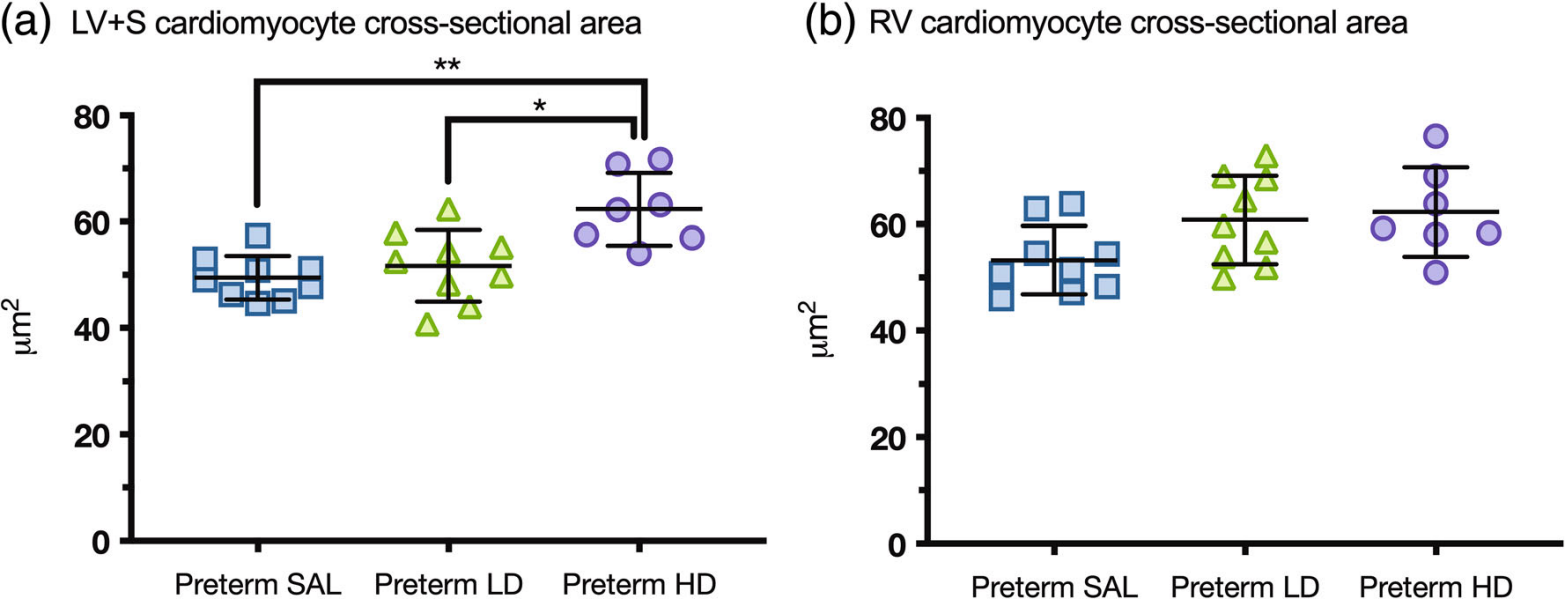
Cardiomyocyte cross‐sectional area. Cardiomyocyte cross‐sectional area was assessed in the left ventricle + septum (LV + S; a) and right ventricle (RV; b) of preterm lambs treated with saline (blue squares, preterm SAL, *n* = 9) or dexamethasone at a low‐dose (green triangles, preterm LD, *n* = 9) or high‐dose (purple circles, Preterm HD, *n* = 7). Data are presented as mean ± *SD*, *p < .05, **p < .001.

Cardiomyocyte cross‐sectional area was measured to assess the effects of postnatal dexamethasone exposure on cardiomyocyte cell size (Figure 6). Preterm lambs treated with high‐dose dexamethasone exhibited an increase in LV + S cardiomyocyte cross‐sectional area compared to preterm lambs treated with low‐dose dexamethasone (*p* = .009) or saline (*p* = .0008). Low‐dose dexamethasone had no effect on LV + S cardiomyocyte cross‐sectional area compared to control preterm lambs. Postnatal dexamethasone exposure had no significant effect on cardiomyocyte cross‐sectional area in the RV: cross‐sectional area trended higher in preterm lambs treated with low‐dose (*p* = .057) and high‐dose (*p* = .063) dexamethasone compared to control preterm lambs.

### 3.8 Gene expression in the LV and RV

The genes examined in the preterm lamb hearts showed different expression between the LV and RV (Table 3). *COL1A1* was significantly downregulated in the LV of preterm lambs treated with low‐dose dexamethasone (*p* < .05) compared to control preterm lambs. However, preterm lambs treated with high‐dose dexamethasone (*p* < .05) showed downregulation of *IGF‐1* in the LV compared to preterm lambs treated with low‐dose dexamethasone or saline. All other genes had similar expression in the LV between all groups. The relative mRNA expression of selected genes involved in inflammation, cardiac remodeling and hypertrophy (*IL‐1b*, *IL‐18*, *MMP9*, *MYH7*, and *NPPA*) were highly variable within groups, including the control group.

**TABLE 3:** Gene expression in the left and right ventricles of preterm lambs treated postnatally with saline (SAL) or low‐dose (LD) or high‐dose (HD) dexamethasone.

Genes of interest	Left ventricle			Right ventricle		
	Preterm SAL	Preterm LD	Preterm HD	Preterm SAL	Preterm LD	Preterm HD
*ATP2A2*	1.00 ± 0.26	0.92 ± 0.27	0.95 ± 0.33	1.00 ± 0.33	1.11 ± 0.36	1.24 ± 0.29
*COL1A1*	1.00 ± 0.40	0.56 ± 0.19*	0.58 ± 0.39	1.00 ± 0.60	1.35 ± 0.92	1.32 ± 0.78
*COL3A1*	1.00 ± 0.59	1.09 ± 0.34	0.56 ± 0.45	1.00 ± 0.46	1.30 ± 0.36	1.12 ± 0.58
*GATA4*	1.00 ± 0.40	0.88 ± 0.30	0.98 ± 0.30	1.00 ± 0.50	1.27 ± 0.40	1.82 ± 0.51*
*IGF‐1*	1.00 ± 0.42	0.76 ± 0.45	0.34 ± 0.12*,** ^**#**^	1.00 ± 0.44	0.97 ± 0.52	0.81 ± 0.41
*IL‐1b*	1.00 ± 3.85	0.78 ± 1.44	0.36 ± 0.19	1.00 ± 2.08	0.90 ± 1.42	0.35 ± 0.25
*IL‐18*	1.00 ± 0.97	1.07 ± 1.21	0.97 ± 0.78	1.00 ± 0.57	0.36 ± 0.28*	0.21 ± 0.22*
*MMP9*	1.00 ± 4.38	1.25 ± 3.24	1.25 ± 1.15	1.00 ± 2.06	0.72 ± 1.13	0.30 ± 0.86
*MYH7*	1.00 ± 1.00	1.01 ± 0.34	0.92 ± 0.44	1.00 ± 0.63	1.44 ± 0.85	1.28 ± 0.45
*NPPA*	1.00 ± 2.57	0.33 ± 0.31	0.95 ± 3.41	1.00 ± 3.79	0.78 ± 1.43	1.31 ± 4.87
*PPARA*	1.00 ± 0.53	0.92 ± 0.23	0.90 ± 0.40	1.00 ± 0.61	0.97 ± 0.28	1.46 ± 0.41
*PPARGC1A*	1.00 ± 0.54	0.82 ± 0.25	0.91 ± 0.40	1.00 ± 0.31	1.09 ± 0.28	1.54 ± 0.47*
*RYR2*	1.00 ± 0.82	1.14 ± 0.65	1.00 ± 0.56	1.00 ± 0.36	1.35 ± 0.36	1.52 ± 0.43*
*SLC8A1*	1.00 ± 0.79	0.74 ± 0.26	0.88 ± 0.52	1.00 ± 0.51	0.93 ± 0.23	1.39 ± 0.47
*TGFB1*	1.00 ± 0.27	0.76 ± 0.18	0.95 ± 0.48	1.00 ± 0.34	1.25 ± 0.36	1.36 ± 0.24
*TIMP2*	1.00 ± 0.38	0.61 ± 0.19	0.87 ± 0.36	1.00 ± 0.54	0.87 ± 0.13	1.54 ± 0.62**
*TLR4*	1.00 ± 0.59	0.91 ± 0.37	0.80 ± 0.23	1.00 ± 0.50	1.07 ± 0.28	0.96 ± 0.35
*TNF*	1.00 ± 0.49	0.45 ± 0.47	0.48 ± 0.30	1.00 ± 0.42	1.34 ± 0.83	1.17 ± 0.66
*VEGFA*	1.00 ± 0.66	0.92 ± 0.42	1.10 ± 0.46	1.00 ± 0.45	1.62 ± 0.79 *	1.88 ± 0.28 *

*Note*: All gene expression was represented as the calibrated normalized relative quantity (CNRQ); normalized to YWHAZ and expressed relative to the preterm saline group normalized at 1.00. Data are presented as mean ± *SD*. Significance accepted at *p* < .05.

* Significant difference compared to preterm SAL group.

** significant difference compared to preterm LD group.

*VEGFA* and *IL‐18* were differentially expressed in the RV of preterm lambs treated with low‐ and high‐dose dexamethasone compared to control preterm lambs (*p* < .05). *GATA4*, *PPARGC1A*, and *RYR2* were upregulated in the RV of preterm lambs treated with high‐dose dexamethasone compared to control preterm lambs. *TIMP2* was upregulated in the RV of preterm lambs treated with high‐dose dexamethasone compared to the low‐dose dexamethasone group but was not different compared to the control group. All other genes analyzed in the RV had no change in relative expression between groups. However, as observed in the LV, relative mRNA expression was highly variable in the RV for selected genes involved in inflammation, cardiac remodeling and hypertrophy (*COL1A1*, *IL‐1b*, *MMP9*, *MYH7*, and *NPPA*).

## 4 DISCUSSION

Postnatal dexamethasone treatment is used in the care of preterm infants who require respiratory support after birth. The findings of the present study in a clinically relevant preterm sheep model, show dose‐related adverse impacts of postnatal dexamethasone treatment on the preterm heart. Dexamethasone therapy at a low‐dose (currently used in the clinical setting) did not affect the growth of the cardiomyocytes, cardiomyocyte endowment or deposition of myocardial collagen but was associated with increased inflammatory infiltrates in the LV + S myocardium. Adverse effects of dexamethasone in the preterm heart were observed with higher doses of dexamethasone: high‐dose dexamethasone induced cardiomyocyte hypertrophy, increased cardiomyocyte cell cycle activity and further exacerbated inflammatory infiltrates within the LV + S, and reduced the complement of cardiomyocytes and markedly altered cardiac gene expression within the RV. Many of the observed cardiac changes associated with high‐dose postnatal dexamethasone treatment may impact neonatal cardiac function as well as influence postnatal growth of the heart and subsequent long‐term cardiac function.

### 4.1 Cardiac growth in response to dexamethasone

In the present study, a dose dependent effect of dexamethasone treatment was clearly evident within the preterm hearts. High‐dose dexamethasone perturbed cardiac growth at 1 week of postnatal life compared to saline‐treated preterm lambs; however, the heart weight was proportional to body weight when adjusted to body weight. The overall impact of high‐dose dexamethasone exposure on cardiac growth in early life (whether directly, or indirectly through effects on body growth) has the potential to influence the normal growth trajectory of the heart, and potentially body growth. In accordance with these findings in the high‐dose dexamethasone group, rat pups treated with dexamethasone, equivalent to a 21‐day tapering dose in humans, show altered cardiac growth in the neonatal period, including reduced heart and LV weight, and reduced LV wall and lumen volume (Adler et al., 2010; Bal et al., 2009; de Vries et al., 2006). In addition to impaired cardiac growth, dexamethasone therapy reduces LV systolic function and increases wall stress in rats (Bal et al., 2005). Collectively, the findings from these previous studies suggest that early postnatal dexamethasone treatment has negative effects on cardiac growth and function. Encouragingly, however, the lack of any impact of low‐dose postnatal dexamethasone on heart size or body growth in this study suggests that dexamethasone dose is likely a contributing factor to the adverse effects reported previously.

### 4.2 Anti‐inflammatory actions of glucocorticoids on the preterm myocardium

Glucocorticoids exhibit potent anti‐inflammatory and immunosuppressive actions and hence are used widely in the neonatal intensive care unit to treat a number of inflammatory conditions, including severe bronchopulmonary dysplasia (Coutinho & Chapman, 2011; Doyle et al., 2017). To the contrary, however, our study shows minimal evidence of dexamethasone exerting anti‐inflammatory effects in the myocardium (except downregulation of IL‐18 in the RV). Importantly, and of concern, there was a high presence of immune cell infiltrates (CD45+ cells, a leukocyte marker) in the LV + S of the preterm lambs treated with high‐ and low‐dose dexamethasone. The presence of immune cell infiltrates appeared to be dose‐related and is likely an acute response to dexamethasone treatment. The observed leukocyte infiltration into the myocardium may be consequently due to neutrophilic leukocytosis, which is another side effect of glucocorticoid treatment observed in preterm infants (Evans et al., 1988; Noble‐Jamieson et al., 1989). However, as the increased infiltration of leukocytes into the myocardium following dexamethasone treatment was observed only in the LV + S, it is possible that the leukocyte migration out of the microvasculature into the LV + S is an inflammatory response, rather than a systemic circulatory effect.

The infiltration of inflammatory cells into the myocardium may trigger the induction of fibrosis (Frangogiannis, 2017; Nielsen et al., 2017). There was no evidence to indicate that dexamethasone treatment triggered maladaptive extracellular remodeling either directly or through the infiltration of inflammatory cells into the myocardium within the first week of life, as myocardial interstitial collagen levels were not changed by dexamethasone treatment. Likewise, gene expression did not increase for any of the examined genes associated with collagen synthesis (*COL1A1* and *COL3A1*). In fact, *COL1A1* was significantly down regulated in the LV myocardium following low‐dose dexamethasone treatment and may maintain normative levels of collagen within the preterm heart.

### 4.3 Cardiomyocyte growth and maturational response to dexamethasone

The inhibition of cellular proliferation by glucocorticoids is well described (Rogatsky et al., 1997). In relation to cardiomyocytes, dexamethasone induces premature terminal differentiation in cultured neonatal cardiomyocytes (Gay et al., 2016) and suppresses proliferation and stimulates premature binucleation of cardiomyocytes, hallmarks of terminal differentiation, in rat pups (de Vries et al., 2006; Gay et al., 2015). Interestingly, antenatal chronic exposure to cortisol in fetal sheep at a similar postconceptional age to this study exhibit augmented cardiomyocyte growth, either through hypertrophic growth of cardiomyocytes in the presence of elevated blood pressure (Lumbers et al., 2005) or increased cardiomyocyte cell cycle activity with no effect on blood pressure (Giraud et al., 2006). We therefore hypothesized that postnatal dexamethasone exposure would accelerate cardiomyocyte maturation accompanied by an inhibition of cardiomyocyte proliferation and increased binucleation. In the sheep heart, it is generally considered that mature cardiomyocytes are binuclear, whereas mononucleated cardiomyocytes are considered to be immature and capable of proliferation (Burrell et al., 2003). Hence, our finding of no accelerated maturation of the preterm heart after dexamethasone exposure was unexpected. Instead, high‐dose dexamethasone exposure led to increased DNA synthesis in LV + S cardiomyocytes. It may be that high‐dose dexamethasone exposure, with no change in mean arterial blood pressure, stimulated LV + S cardiomyocyte proliferation and/or induced polyploidy, a well‐described stress‐response in cardiomyocytes whereby cardiomyocytes undergo DNA synthesis without karyokinesis or cytokinesis (Liu et al., 2010; Ovrebo & Edgar, 2018).

The reduced complement of cardiomyocytes in the RV in preterm lambs treated with high‐dose dexamethasone is of concern, given that cardiomyocytes have a limited capacity to proliferate once they have undergone the maturational transition in early life (Clubb Jr. & Bishop, 1984; Thornburg et al., 2011). These RV cardiomyocyte deficits remained even when adjusting for body weight in the high‐dose group. The lack of effect of dexamethasone on LV + S cardiomyocyte complement, despite‐reduced RV cardiomyocyte complement, is intriguing. The ventricular differences imply that the negative effects of dexamethasone treatment on RV endowment are an indirect effect (perhaps in relation to pulmonary haemodynamics) rather than a direct effect on the cardiomyocytes.

A reduced complement of cardiomyocytes at the beginning of life has the potential to adversely impact the adaptive and functional capabilities of the RV leading to myocardial dysfunction in later life. In fact, young adults born preterm respond to physical exercise with impaired LV and RV function suggestive of a reduced cardiomyocyte reserve (Goss et al., 2018; Huckstep et al., 2018; Huckstep et al., 2021). Furthermore, cardiovascular imaging highlights a unique cardiac geometry in young adults who were born preterm, and the impact of preterm birth is greatest in the RV, evidenced by a shorter ventricle with greater ventricular mass and myocardial dysfunction (Lewandowski et al., 2013a; Lewandowski et al., 2013b). Interestingly, a proportion of the variation in RV mass in young adults who were born preterm was attributed to postnatal ventilation (Lewandowski, Bradlow, et al., 2013), which is of relevance given that postnatal ventilation is often accompanied with postnatal steroid treatment. It is therefore important in future studies to include the use of postnatal steroids as a factor, when examining the altered postnatal growth of the preterm heart.

### 4.4 Dexamethasone‐induced cardiomyocyte hypertrophy

The condition of hypertrophic cardiomyopathy experienced by preterm infants undergoing dexamethasone therapy (Evans, 1994; Gill et al., 1996; Skelton et al., 1998; Werner et al., 1992; Zecca et al., 2001) is histologically identified by irregular cardiomyocyte hypertrophy and disarray, interstitial fibrosis, and microvasculature disease (Davies & McKenna, 1995; Lombardi & Betocchi, 2002). Although no differences in interstitial collagen were detected in the present study, there was induction of cardiomyocyte hypertrophy (highly significant increase in cardiomyocyte cross‐sectional area) in the LV + S of preterm lambs exposed to the high‐dose of dexamethasone. However, genes associated with cardiac hypertrophy, including *GATA4*, *MYH7*, and *NPPA*, were not differentially expressed in the LV. In support of these cardiomyocyte hypertrophy findings, previous preclinical studies have demonstrated that dexamethasone exposure induces cardiomyocyte hypertrophy (de Vries et al., 2006; Riede et al., 2001) that normalizes within a short period following cessation of dexamethasone treatment (de Vries et al., 2006) but reappears in adulthood (Bal et al., 2009; De Vries et al., 2002). However, whether the effects of postnatal dexamethasone on cardiac hypertrophy are transient or lead to permanent changes in myocardial structure, needs further investigation.

### 4.5 Limitations

All preterm lambs were delivered at a gestational age where they required antenatal and postnatal care for their survival. In this regard, all preterm lambs were exposed to antenatal glucocorticoids (maternal betamethasone) before preterm delivery, which may have influenced cardiac development. In addition, the postnatal care of preterm lambs was managed pragmatically on an individual basis due to their requirements. Individualized care may contribute to variability within preterm groups. The preclinical study design randomly assigned postnatal treatment in a blinded manner. Consequently, preterm lambs were not allocated to specific treatment groups based on sex, and due to uneven numbers of males and females within groups we did not analyze sex differences. The outcomes of the present study were short term and therefore we are unable to comment if the cardiac changes persist long term.

## 5 CONCLUSION

There is clinical interest in modifying postnatal steroid use to maximize the benefits of potent steroids, such as dexamethasone, while minimizing the adverse side effects, for the safer clinical management of preterm infants. We conclude that neither a high‐ or low‐dose of dexamethasone exposure is devoid of adverse impacts in the neonatal preterm heart with the LV and RV differentially affected in a dose‐dependent manner. Follow‐up studies into the adult cardiovascular health of those who were exposed to postnatal steroids as a preterm infant have not been conducted and such studies are warranted. Although, high‐dose dexamethasone is no longer commonly used in the clinic, a number of individuals who were affected by this regimen as a preterm infant are now in adulthood with unknown cardiovascular sequelae.

## AUTHOR CONTRIBUTIONS

**Amanda Vrselja:** Conceptualization; data curation; formal analysis; investigation; methodology; project administration; resources; software; validation; visualization; writing – original draft; writing – review and editing. **Jennifer Jane Pillow:** Conceptualization; data curation; formal analysis; funding acquisition; investigation; methodology; project administration; resources; software; supervision; validation; visualization; writing – review and editing. **Jonathan G. Bensley:** Conceptualization; data curation; formal analysis; investigation; methodology; resources; software; validation; writing – review and editing. **Siavash Ahmadi‐Noorbakhsh:** Data curation; investigation; methodology; project administration; writing – review and editing. **Peter B. Noble:** Conceptualization; data curation; formal analysis; funding acquisition; investigation; methodology; project administration; resources; validation; writing – review and editing. **Mary Jane Black:** Conceptualization; data curation; funding acquisition; investigation; methodology; project administration; resources; software; supervision; validation; visualization; writing – review and editing.

## FUNDING INFORMATION

This work was supported by a Telethon Perth Children's Hospital Research Fund project grant, and a National Health and Medical Research Council (NHMRC) project grant (GNT1057759) and Centre of Research Excellence (GNT1057514). Amanda Vrselja was supported by a MBio Postgraduate Discovery Scholarship. Jennifer Jane Pillow was a recipient of a National Health and Medical Research Council Senior Research Fellowship (RF1077691). Equipment and consumable support was provided through unrestricted grants from Fisher and Paykel Healthcare (ventilation circuits), Chiesi Farmaceutici S.p.A. (poractant alfa), and ICU Medical (monitoring lines).

## CONFLICT OF INTEREST STATEMENT

The authors declare no conflict of interest. The funders had no role in the design of the study; in the collection, analyses, or interpretation of data; in the writing of the manuscript, or in the decision to publish the results.
